# Amygdala Persistence in Aging: Replication and Extension of Puccetti et al. (2021)

**DOI:** 10.64898/2026.10.14.759196

**Published:** 2026-10-20

**Authors:** Adam Turnbull, Meishan Ai, James J. Gross, Feng Vankee-Lin

## Abstract

Age-related differences in affect are well established, but their neural mechanisms remain unclear. In a preregistered replication of Puccetti et al. (2021) and extension, we examined age, affect, left-amygdala negative persistence, and amygdala–ventromedial prefrontal cortex (vmPFC) task-based connectivity using MIDUS 3 daily diary and fMRI data. Older age predicted higher positive and lower negative affect in the daily diary sample ( *N* = 1,168; *ps* < .001). In the primary diary + fMRI sample ( *N* = 81; ages 48–95, 61.7% female, 82.7% White), greater persistence predicted higher raw negative affect (β = 0.278, 95% CI [0.012, 0.544], *p* = .020); associations with positive and log-transformed negative affect were nonsignificant ( *ps* ≥ .063). Older age predicted lower persistence in the final fMRI sample ( *N* = 127; β = −0.00260, 95% CI [−0.00504, −0.00016], *p* = .018), but this association was weaker in the primary diary + fMRI sample ( *p* = .200), with no evidence of mediation. Greater anterior vmPFC connectivity predicted lower raw negative affect (β = −0.106, 95% CI [−0.223, 0.012], *p* = .039) and log-transformed negative affect ( *p* = .014). In MIDUS Refresher 1, affect estimates were directionally similar but nonsignificant ( *N* = 48; *ps* ≥ .081), whereas the age–persistence estimate reversed direction. These findings provide partial support for amygdala persistence and task-based connectivity as neural correlates of daily affect, while highlighting variability in the magnitude and replicability of fMRI–behavior associations.

**Public Significance Statement:**

Among middle-aged and older adults, lingering brain responses to negative images and communication between emotion-related brain regions were associated with negative feelings in daily life. However, the findings did not establish why emotional experience differs with age, and evidence from a second sample was inconclusive. Larger studies are needed to determine how reliably these brain measures relate to everyday emotional well-being.

## Full Text Availability

The license terms selected by the author(s) for this preprint version do not permit archiving in PMC. The full text is available from the preprint server.

